# Supplementary material for: Magnetic fluctuations driven insulator-to-metal transition in Ca(Ir1−xRux)O3
Source: Sci Rep. 2015 Dec 9;5:18047. doi: 10.1038/srep18047 (PMC4673448; doi:10.1038/srep18047)
Supplement: Supplementary Information [file srep18047-s1.pdf]

Supplementary Material: Magnetic fluctuations driven insulator-to-metal transition in $Ca(Ir_{1-x}Ru_x)O_3$

J. Gunasekera[1], L. Harriger[2], A. Dahal[1], T. Heitmann[3], G. Vignale[1], and D.K. Singh[1]

[1]*Department of Physics and Astronomy, University of Missouri, Columbia, MO 65211,USA*

[2]*NIST Center for Neutron Research, Gaithersburg, MD 20899, USA and*

[3]*University of Missouri Research Reactor, University of Missouri, Columbia, MO 65211, USA*

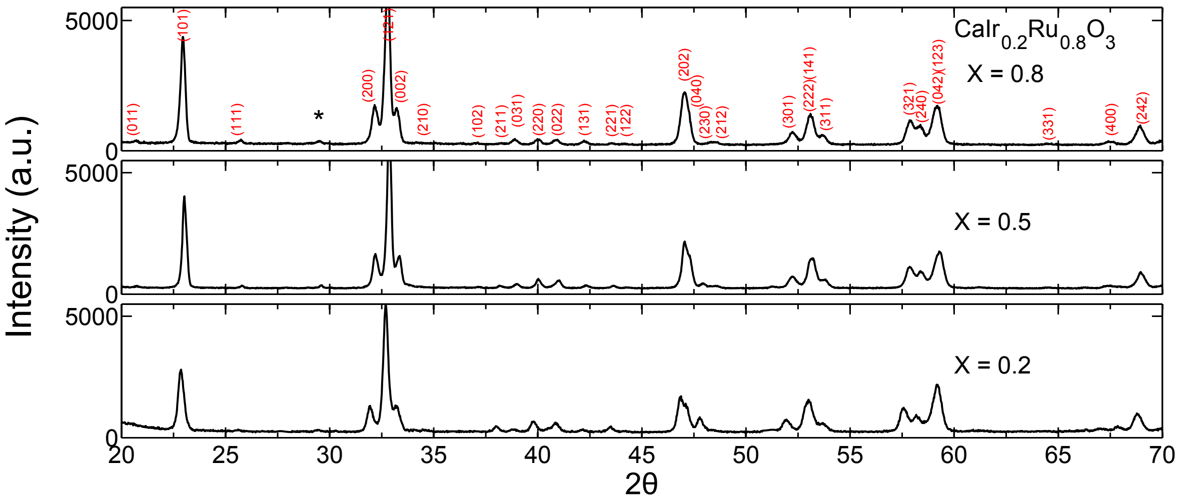

FIG. S1: **Xray diffraction pattern of chemically doped Ca(Ir$_{1-x}$Ru$_x$)O$_3$. a-c,** The polycrystalline samples were characterized using powder Xray diffraction method. The powder diffraction data is refined using the commercially available JADE software. The high purity of the sample is evident from the XRD data, where every single peak, except one small peak at 29° as marked by the asterisk, is identified with a crystallographic wave vector of the orthorhombic structure. The small peak with asterisk, with less than 0.2 percent intensity compared to the largest peak, is found in every sample measured on the XRD system; leading us to consider that it may be arising due the sample holder. A small shift in the peak positions, as the composition x varies, is attributed to the chemical substitution of Ru by Ir.

The high purity polycrystalline samples of Ca(Ir$_{1-x}$Ru$_x$)O$_3$ were synthesized by conventional solid state reaction method using ultra-pure ingredients of IrO_2, RuO_2 and $CaCO_3$. Starting materials were mixed in stoichiometric composition, with five percent extra RuO_2 to compensate for their rapid evaporation (in Ru-doped perovskites), pelletized and sintered at 950° for three days. The furnace cooled samples were grinded, pelletized and sintered at 1000° for another three days. Resulting samples were characterized using Siemens D500[1] powder X-ray diffractometer, confirming the single phase of material, see Fig S1. The X-ray diffraction data were analyzed using a widely used commercial software JADE.[1]

Next, we have investigated the presence of magnetic order in few chemical compositions of CaIr$_{1-x}$Ru$_x$O$_3$ using detailed elastic neutron scattering measurements. Two chemical compositions of x = 0.8 and x = 1 were used for that purpose. Strong neutron absorbing nature of iridium make the measurement and the quantitative analysis very difficult in low

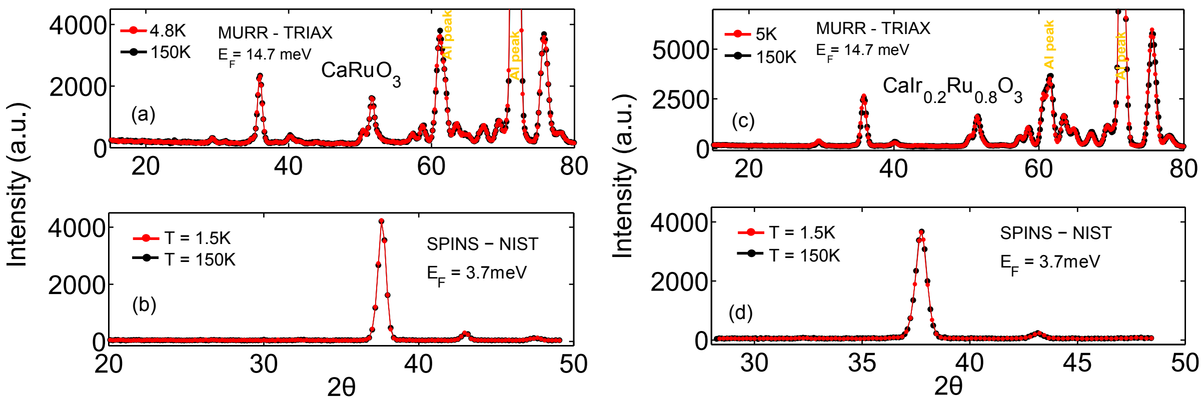

FIG. S2: **Elastic neutron scattering measurements on different compositions**. **a,** Elastic neutron scattering measurements on x = 1 composition were performed on a thermal triple axis spectrometer TRIAX with E_i=E_f=14.7 meV at the Missouri University Research Reactor. Observed sharp peaks in this figure are attributed to the aluminum sample can and the nuclear structure factor. The difference between the elastic scans data at 4.8 K and 200 K is found to be statistically insignificant. Hence, no evidence of any magnetic order is detected. **b,** High resolution elastic measurements were performed at the SPINS cold triple axis spectrometer at the NIST Center for Neutron Research. No trace of magnetic order, as evidenced by the statistically insignificant difference in neutron counts at low (1.5 K) and high temperature data (150 K), is detected. **c-d,** Similar measurements were performed on x = 0.8 chemical composition on TRIAX (fig. 2c) and SPINS (fig. 2d) spectrometers. Once again, no trace of a magnetic order is detected. Error bar represents one standard deviation in all figures.

x compositions that have higher iridium concentrations. Elastic measurements, 2θ-scans, were carried out on a thermal triple-axis spectrometer TRIAX at the Missouri University Research Reactor and on a cold triple axis spectrometer, SPINS, at the NIST Center for Neutron Research. Measurements on the thermal spectrometer was performed with a fixed final energy of 14.7 meV and the collimation setting of 60'-PG (pyrolitic graphite) filter-monochromator-PG (pyrolitic graphite) filter-80'-sample-40$'$-PG filter- PG analyzer-80'-detector. Measurements on SPINS were performed at the fixed final energy of E_F = 3.7 meV with the collimators sequence of PG (mono)-Be Filter-Sample-BeO filter-80$'$-flat analyzer-Detector. As shown in Fig S2, no change in the peak intensities are observed at different temperatures in both compositions. These measurements suggest the absence of magnetic order in these compounds. While this assessment is consistent with previous re-

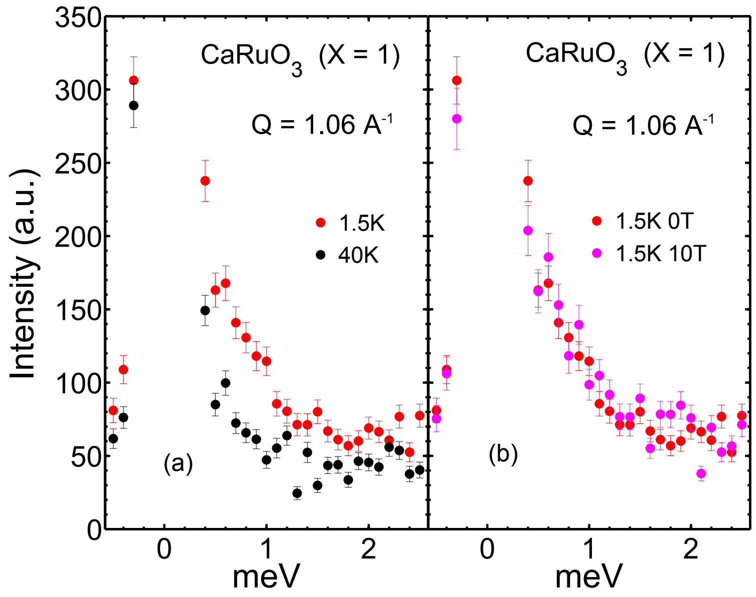

FIG. S3: **Inelastic neutron scattering measurements on x = 1 composition at different temperatures and fields**. **a,** Inelastic measurements show that the neutron intensity at low energy (up to $E \simeq 2$ meV) is significantly higher at low temperature. Higher spectral count at low temperature at $H = 0$ T confirm the presence of the dynamic magnetism in this system. **b,** Unlike the temperature dependence of the dynamic spectral weight, field application up to $H = 10$ T does not have any effect on the inelastic magnetic properties. Error bar represents one standard deviation in all figures.

ports on $CaRuO_3$ (x = 1),[2, 3] this is the first time we have performed detailed elastic measurements on x = 0.8 composition.

The temperature and magnetic field dependences of the dynamic properties were also investigated in these two compositions (x = 1 and x = 0.8) of $Ca(Ir_{1-x}Ru_x)O_3$ using inelastic neutron scattering measurements on the SPINS cold triple-axis spectrometer. The spectrometer's configuration for inelastic measurements was PG (mono)-80'-Sample-BeO filter-radial collimator-11 blades focused analyzer-Detector. The spectrometer's resolution was determined using an empty vanadium can scan. At $E_F = 3.7$ meV, the spectrometer's resolution was determined to be $\simeq 0.16$ meV. The characteristic scans at different temperatures and fields are plotted in Fig. S3 and Fig. S4. As we can see in Fig.S3a, the low energy ($E \leq 2$ meV) spectral weight of the dynamic structure factor in x = 1 composition is significantly higher at $T = 1.5$ K and $H = 0$ T, compared to the spectral weight at $T =$ 40 K. At $H = 10$ T, no difference in the neutron intensity count, compared to the intensity

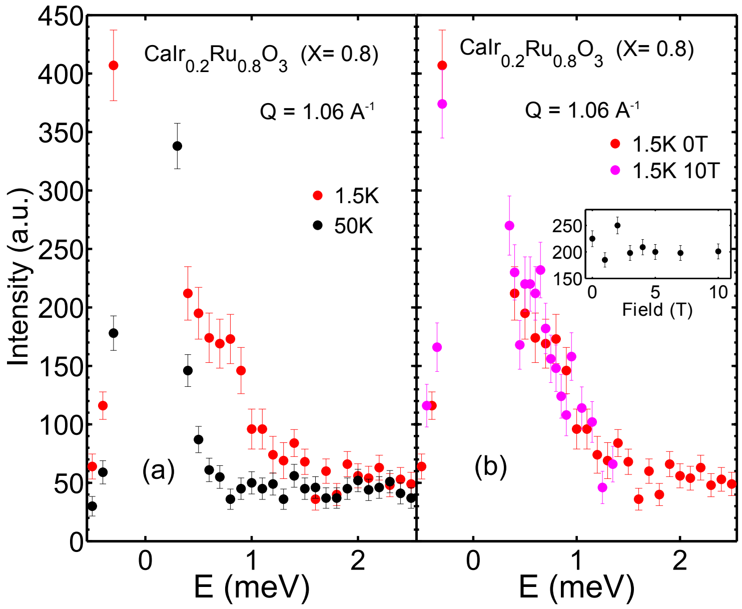

FIG. S4: **Inelastic neutron scattering measurements on x = 0.8 composition at different temperatures and fields**. **a,** Similar to the x = 1 composition, the higher spectral count at low temperature at $H = 0$ T confirm the presence of the dynamic magnetism in this system. **b,** Once again, the field application up to $H = 10$ T does not have any effect on the inelastic magnetic properties. Error bar represents one standard deviation in all figures.

at $H = 0$ T, is detected. These measurements further confirm the presence of magnetic fluctuations at low temperature. We also note that the electrical resistivity as a function of temperature also exhibits field-independent character across the group in $Ca(Ir_{1-x}Ru_x)O_3$. The field independence of inelastic intensity, depicting magnetic fluctuations, is related to the electrical properties in that regard. Similar dynamic magentic behaviors are observed in x = 0.8 composition, as shown in Fig. S4a and S4b.

---

[1] National Institute of Standards and Technology or the Department of Commerce does not endorse this commercial product.

[2] G. Cao *et al.*, *Solid State Commun.* **148**, 305 (2008).

[3] T. He and R. J. Cava, *J. Phys.: Cond. Matt.* **13**, 8347 (2001).
